# A study of the personality trait-focused digital mental health intervention

**DOI:** 10.1192/j.eurpsy.2024.1152

**Published:** 2024-08-27

**Authors:** S. Jeong, H. Kim, S. K. Lho, S. Mun, I. Hwang, S. Kim, H. Lim, H. Kim, W. Moon, M.-S. Shin

**Affiliations:** ^1^Psychiatry, Seoul National University Hospital; ^2^ 40FY inc; ^3^Seoul National University College of Medicine, Seoul, Korea, Republic Of

## Abstract

**Introduction:**

Mental healthcare services that address a variety of primary complaints which are highly related to maladaptive personality traits among the general population are important to prevent developing psychiatric disorders.

**Objectives:**

This study aimed to examine the effectiveness of a digital mental health service (named “Mindling”) that focuses on maladaptive personality traits in the general population.

**Methods:**

Participants were recruited through a South Korean community website and screened for adults between the ages of 18 and 60 in terms of personality traits such as perfectionism, low self-esteem, social isolation, or anxiety. Participants were allocated to four intervention programs (Riggy, Pleaser, Shelly, and Jumpy) based on their screening results and were randomly assigned to digital treatment and waitlist groups. Each intervention program was conducted online for 10 weeks. The primary outcomes were all measured by self-report questionnaires; in addition to stress levels, each program included measures of perfectionism (Riggy), low self-esteem (Pleaser), loneliness (Shelly), and anxiety (Jumpy). The secondary outcomes included self-efficacy, depression, and other psychological states. All participants completed pre-treatment (baseline), intervention (week 5), and post-treatment (week 10) assessments, and the treatment group completed a separate follow-up assessment (week 14).

**Results:**

In the treatment group, 70.05% of the participants completed the full course of the digital intervention. The mean scores for each primary outcome measure and some secondary outcome measures were significantly different between baseline and post-treatment in the treatment group for the Total, Riggy, Pleaser, Shelly, and Jumpy programs, but these differences were not observed in the waitlist group. In addition, mean differences between the treatment and waitlist groups at post-treatment assessment were significant for all primary outcome measures and some secondary outcome measures. Specifically, the levels of stress (Total program), perfectionism (Riggy), loneliness (Shelly), and anxiety (Jumpy) were significantly lower in the treatment group, while self-esteem (Pleaser) was higher. In addition, the mean differences between post-treatment and follow-up assessment data were not statistically significant for all primary outcome measures and nearly all secondary outcome measures.

**Conclusions:**

This study validated the effectiveness of the digital intervention program targeting maladaptive personality traits and suggested its sustainable effects.

**Disclosure of Interest:**

None Declared

